# Effects of Background Fluid on the Efficiency of Inactivating Yeast with Non-Thermal Atmospheric Pressure Plasma

**DOI:** 10.1371/journal.pone.0066231

**Published:** 2013-06-14

**Authors:** Young-Hyo Ryu, Yong-Hee Kim, Jin-Young Lee, Gun-Bo Shim, Han-Sup Uhm, Gyungsoon Park, Eun Ha Choi

**Affiliations:** Plasma Bioscience Research Center, Kwangwoon University, Seoul, Korea; Seoul National University, Republic of Korea

## Abstract

Non-thermal plasma at atmospheric pressure has been actively applied to sterilization. However, its efficiency for inactivating microorganisms often varies depending on microbial species and environments surrounding the microorganisms. We investigated the influence of environmental factors (surrounding media) on the efficiency of microbial inactivation by plasma using an eukaryotic model microbe, *Saccharomyces cerevisiae*, to elucidate the mechanisms for differential efficiency of sterilization by plasma. Yeast cells treated with plasma in water showed the most severe damage in viability and cell morphology as well as damage to membrane lipids, and genomic DNA. Cells in saline were less damaged compared to those in water, and those in YPD (Yeast extract, Peptone, Dextrose) were least impaired. *HOG1* mitogen activated protein kinase was activated in cells exposed to plasma in water and saline. Inactivation of yeast cells in water and saline was due to the acidification of the solutions by plasma, but higher survival of yeast cells treated in saline may have resulted from the additional effect related to salt strength. Levels of hydroxyl radical (OH**^.^**) produced by plasma were the highest in water and the lowest in YPD. This may have resulted in differential inactivation of yeast cells in water, saline, and YPD by plasma. Taken together, our data suggest that the surrounding media (environment) can crucially affect the outcomes of yeast cell plasma treatment because plasma modulates vital properties of media, and the toxic nature of plasma can also be altered by the surrounding media.

## Introduction

Reactive oxygen species (ROS) and reactive nitrogen species (RNS) have lethal effects on microorganisms [1]. Phagocytic cells in the immune system synthesize various ROS, RNS, and intermediates upon infection with pathogenic microbes, and the toxicity of these reactive species has been well demonstrated in many studies [1]–[2]. Reactive species are a good means for attacking pathogenic microorganisms, and effective use of them provides a promising tool for controlling microbial diseases. To date, chemicals and materials producing ROS and RNS or inducing endogenous production of ROS and RNS have been frequently used but their antimicrobial effects are controversial [3]–[4]. As an alternative control tool, non-thermal plasma at atmospheric pressure developed in physics has been actively applied and examined for its effectiveness in microbial inactivation [5]–[7]. Plasma, as a mixture of ionized gas, produces various ROS and RNS. These reactive species are thought to be the major components that have antimicrobial effects, but UV, ions, and the electric field from plasma are also involved in killing microorganisms [8].

The efficiency of plasma in inactivating microorganisms can be regulated by modulating the physical conditions of plasma discharge such as electrical power, fed gases, and exposure time [9]–[11]. However, recent studies have also demonstrated a broad spectrum of inactivation efficiency of plasma in various microbial species and environments surrounding microorganisms. Prokaryotic (bacteria) and eukaryotic (yeast and fungi) microbes have different susceptibility to plasma, and, in many cases, prokaryotic microbes are more vulnerable to plasma [10], [12]–[17]. Plasma can produce different control outcomes to the same microbial species, depending on the surrounding environment. One study demonstrated that bacteria present in various physiological fluids are killed by plasma at different rates and that the different death rates are probably caused by differential changes in components and ions in the solutions caused by plasma [18]. In addition, plasma is more effective in eradicating bacteria on non-biological surfaces than on skin [19]. It may be possible that applying plasma technology to natural environments is more complicated than we expect. Therefore, there is a great need to clarify the mechanisms regulating efficiency of microbial inactivation by plasma to improve the effectiveness of plasma during sterilization.

Mechanisms for varying sterilization efficiency have not been studied frequently. Various antimicrobial outcomes can result because environments surrounding microorganisms can respond differently to plasma. Many studies have revealed that plasma changes pH of water to become more acidic and this may affect microbial mortality [20]–[21]. Plasma changes the ion composition of solutions where microbes are submerged because many reactive and excited species produced by the plasma can react with component molecules in solutions [18]. Environments surrounding microorganisms usually provide elements that are crucially needed for microbial survival and propagation such as water, pH, nutrients, osmotic stability, and temperature. The properties of these elements are modulated by plasma through interaction. However, the interaction of plasma with the environment may also alter certain characteristics of plasma such as composition and level of reactive species. In addition, many reactive oxygen and nitrogen intermediates can be generated as a result of reaction between plasma and environmental factors. Despite its importance in elucidating mechanisms for sterilization by plasma, information on the interaction between plasma and the environment is very limited.

In this study, we investigated the impact of environmental factors on the efficiency of plasma in inactivating microbes to understand the mechanism basis for differential efficiency of plasma sterilization. Biological responses of a model microorganism for eukaryotes, *S. cerevisiae* (budding yeast), were analyzed when yeast cells were exposed to an argon (Ar) plasma jet in three different solutions (environments), water, saline and yeast extract peptone dextrose (YPD) media. These three background solutions have different properties with respect to buffering capacity (pH control), osmotic stability, salt strength, and chemical composition. Besides cell responses, the influences of these three solutions on plasma properties were also examined. Our results suggest that a broad spectrum of efficiency for inactivating yeast is possible by modulating the properties of solutions with plasma and that the level of reactive species is affected by the interactions between plasma and the solutions.

## Materials and Methods

### Preparation of Yeast Cells

Budding yeast, *S. cerevisiae* (AH109), was used in the study. Two or three colonies from an *S. cerevisiae* culture plate were suspended in 500 µl of YPD liquid media, and then 100 µl of suspended yeast was inoculated into 15 ml of YPD liquid placed in a 50 ml centrifuge tube. The tubes were incubated at 30°C with shaking for 16 h and then centrifuged at 7,000 rpm for 5 min to harvest the yeast cells. After centrifugation, the liquid layer was discarded, and the cells were resuspended in 6 ml of sterile water. Cell numbers were counted and then cells were divided into three tubes. The tubes were centrifuged at 7,000 rpm for 5 min., and the cells were resuspended in an appropriate volume of each solution (water, saline, or YPD) to obtain 2×10^7^ cells/ml.

### Exposure of Yeast Cells to Ar Plasma Jet

A non-thermal Ar plasma jet at atmospheric pressure was used in the study. Its structure and exposure condition have been described previously [21]. Yeast cells (2×10^7^ cells) seeded in 1 ml of water, saline, or YPD were placed in each well of a 48 well-microtiter plate. The Ar plasma jet was applied to each well for 30 sec, 1 min, and 3 min. The distance between the tip of the needle (inner electrode) and the surface of the microtiter plate was 1.7 cm and the conditions for plasma breakdown were 4 kV voltage, 13 mA current, 22 kHz repetition rate, and 0.4 liter/min of Ar gas flow controlled by a flow meter (Kojima Inst. Inc., Kyoto, Japan). After exposure to plasma, the treated cells were transferred to a new 1.5 ml microfuge tube and pelleted down by centrifugation at 7000 rpm for 5 min. The liquid was removed and then 1 ml of new solutions (water, saline, or YPD) was added in the tube. Cells were resuspended in the new solution and used for further analysis.

### Test for Cell Viability and Structures

Yeast cell viability was assessed by colony forming unit (CFU) number or the 3-(4,5-dimethylthiazol-2-yl)-2,5-diphenyl tetrazolium bromide (MTT) assay after plasma treatment. Yeast cells (2×10^7^) submerged in the three background solutions (water, saline, and YPD) were exposed to plasma or Ar gas (control) for the indicated time and then used for colony formation and the MTT assay [22]. The cell suspension was diluted 10^4^ times and 100 µl was spread onto a YPD agar plate. The plates were incubated at 30°C for 2 days, and the number of colonies was counted. For the MTT assay, 200 µl of cell suspension (2×10^7^ cells) was placed in a microcentrifuge tube and 30 µl of MTT (5 mg/ml; Sigma, St. Louis, MO, USA) was added to each tube. After incubating the tubes at room temperature for 3 hours, they were centrifuged at 10,000 rpm for 2 min. After removing the supernatant, the cells were washed with 1× phosphate buffered saline (PBS) once and 70 µl of DMSO was added to each tube. The tube was incubated at room temperature for 10 min, and then the contents in each tube were transferred to a new 96 well plate. The plate was read at 490 nm with a Synergy™ HT Multi-mode Microplate Reader (BioTek, Winooski, VT, USA).

Scanning (SEM) and transmission (TEM) electron microscopic analyses were performed to examine the morphology and inner structure of the yeast cells. Four treatments with Ar gas (control) or plasma were combined for preparing the samples. Cells exposed to plasma or Ar gas were fixed in 1 ml of Karnovsky’s fixative (2% paraformaldehyde and 2% glutaraldehyde) overnight [23]. The cells were then washed with 1× PBS three times at 4°C for 10 min. each. After the final wash, the cells were fixed again in 1 ml of 1% osmium tetroxide (in 1× PBS) at 4°C for 2 h. The cells were briefly washed with 1× PBS twice at room temperature and then dehydrated. Dehydration was performed by sequential incubation of cells in 1 ml of 30, 50, 70, 80, 90, and 100% (three times) ethanol for 10 min (at each ethanol concentration). For the SEM analysis, 1 ml of 100% hexamethyldisilazane was added to dehydrated cells and incubated at room temperature for 15 min. After repeating this drying step, the specimen was mounted on carbon tape and examined by SEM (JSM 7001F, JEOL, Tokyo, Japan). For the TEM analysis, dehydrated cells were treated with 1 ml of 100% propylene oxide twice at room temperature for 15 min. Then, a mixture (1∶1, v/v) of propylene oxide and Spurr’s resin (eponate [glycerol 12 resin]: DDSA [dodecenyl succinic anhydride]: NMA [methylnadic anhydride]: DMP-30 [(2,4,6-Tris(dimethyl aminomethyl) phenol] = 29∶16:14.3∶0.8) was added to the tubes containing cells, and the tubes were incubated at room temperature with gentle inverting for 2 h. After the incubation, the cells were centrifuged at 10,000 rpm for 5 min and the resin mixture was removed. New Spurr’s resin (1 ml) was added to the tube, and the tube was gently inverted overnight. The next day, cells were centrifuged at 10,000 rpm for 5 min and the resin was removed. New Spurr’s resin was added to the tube, and the tube was inverted for 2 h. Then, tube was moved to 70°C for polymerization and the polymerized resin block (containing cells) was used for micro-sectioning and TEM (JEM 1010, JEOL).

### Lipid Peroxidation Assay

Membrane lipid peroxidation in yeast cells was assessed by monitoring the production of 4 hydroxynonenal (4-HNE) [24]. Yeast cells harvested from 16 h cultures and suspended in water, saline, and YPD (2×10^7^/ml) were treated with Ar gas (control) and plasma for 3 min, and washed with 1× PBS. Treated cells were incubated in 200 µl of anti-mouse 4 4-HNE antibody (Abcam, Cambridge, MA, USA) diluted in 1× PBS (1∶50 dilution) overnight at 4°C. The next day, the cells were washed with 1× PBS twice to remove excess antibody and incubated in 200 µl of anti-mouse IgG-Atto 488 fluorescent secondary antibody (500/522 nm ex./em., Sigma) diluted in 1× PBS (1∶250 dilution) for 3 h at room temperature. Then, cells were washed twice with 1× PBS, resuspended in new PBS, and read at 500/522 nm. Cells were also examined under a Nikon Eclipse Ti-E inverted fluorescence microscope (Nikon, Tokyo, Japan).

### Genomic DNA Extraction and Western Blot Analysis

Yeast cells combined from the four treatments (2×10^7^ cells/treatment) in each background solution were subjected to genomic DNA extraction. Genomic DNA was extracted following a standard molecular biology protocol and resuspended in 50 µl water [25]. The same amount of genomic DNA (2 µg) extracted from cells treated in water, saline, and YPD was loaded on a 1% agarose gel and run for 1 h. Then, the DNA bands were photographed after staining with ethidium bromide.

For Western blot analysis of mitogen activated protein (MAP) kinases, 10^8^ yeast cells in water, saline, and YPD media were treated with plasma or Ar gas (control) for 2 min. The Cells from the two treatment sets were used for total protein extraction, as described previously [26]. Aliquots containing 50 µg protein were resolved on 12% sodium dodecyl sulfate polyacrylamide gel electrophoresis gels which were then blotted onto nitrocellulose membranes. The membranes were blocked in a solution containing 10 mM Tris-Cl, pH 8.0, 150 mM NaCl and 0.05% Tween 20 (TBST) with 5% BSA for 3 h at room temperature. Then, the membrane was incubated with p44/42 MAP kinase (Cell Signaling Technology, Danvers, MA, USA), *HOG1* MAP kinase (Abcam), phospho-p44/42 MAP kinase (Cell Signaling Technology), phospho-p38 MAP kinase (Cell Signaling Technology), and actin (Abcam) antibodies in blocking solution overnight with gentle shaking at 4°C. Actin was used as the loading control. After the treatment with primary antibody, the membrane was washed three times with TBST and then incubated with anti-rabbit IgG conjugated to horseradish peroxidase in blocking solution (1∶5,000) (Cell Signaling Technology) for 3 h at room temperature. The membrane was then washed three times with TBST, treated with SuperSignal West Pico Chemiluminescent substrate (Pierce, Rockford, IL, USA) and imaged using a Vilver imaging system (Vilver, Upland, CA, USA).

### pH Measurement

Water, saline, and YPD liquid in a 48 well plate (1 ml per well) were exposed to Ar gas (control) or plasma for 10, 30, 60, and 180 seconds. After exposure, the pH of each liquid was measured using pH meter (Eutech Instruments, Singapore). All measurements were carried out in triplicate.

### Measurement of ROS and RNS on the Solution Surface, and Inside the Solution and Cells

ROS and RNS levels on the surface of each solution (water, saline, YPD) were analyzed by optical emission spectroscopy (OES). During plasma exposure, the optical emission spectrum was acquired at 1 mm above the surface of each solution.

The three solutions (water, saline, YPD) in a 48 well plate (1 ml per well) were exposed to plasma or Ar gas (control) for 3 min, and then the concentrations of H_2_O_2_ and nitric oxide (NO) were measured using an Amplex H_2_O_2_ assay kit (Invitrogen, Grand Island, NY, USA) and NO detection assay kit (Biovision, Milpitas, CA, USA), respectively, following the manufacturer’s protocols. The OH radical was measured in the solutions as described previously [27]. Each solution (1 ml) containing 20 mM terephthalic acid (Sigma) was exposed to Ar gas (control) or plasma for 3 min, and the fluorescence emitted from hydroxyterephthalic acid (oxidized terephthalic acid by hydroxyl radical) was measured at 310/425 (ex./em.) nm after treatment.

H_2_DCFDA (2′,7′-dichlorodihydrofluorescein diacetate; Invitrogen) and DAF-FM DA (4-amino-5-methylamino- 2′,7′-difluorofluorescein diacetate; Invitrogen**)** were used for detecting ROS and NO, respectively. Yeast cells submerged in water, saline, and YPD (2×10^7^ cells) were treated with Ar gas (control) and plasma for 3 min and then transferred to a microcentrifuge tube. The cells were washed with 1× PBS and 500 µl of 10 µM H_2_DCFDA or DAF-FM DA was added. After a 30°C incubation for 1 h, the cells were washed again with 1× PBS, twice. Then, the cells were recovered in 1× PBS at 30°C for 30 min and read at 495/515 (ex./em.) nm using a microplate reader.

## Results

### Viability, Morphology, and Internal Structure of Yeast Cells in Water, Saline, and YPD

**Figure 1:**
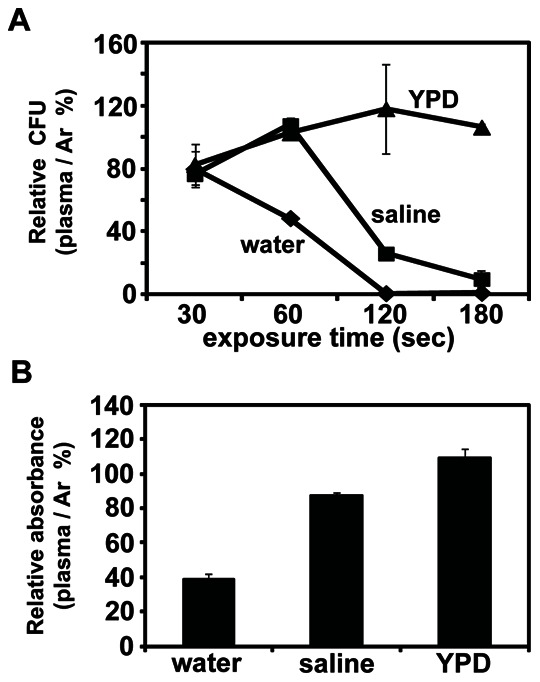
Viability of yeast cells measured by colony formation and MTT assay after plasma treatment. (**A**) Relative percentage of colony formations by yeast cells in water, saline, and YPD after treatment with plasma, compared to Ar gas only. Relative colony forming units (CFU) are calculated as follows: relative CFU (%) = (CFU number of plasma treated yeast/CFU number of Ar gas treated yeast)×100. All value are the average of three technical replicates. (**B**) The relative level of formazan product measured as absorbance at 490 nm in cells treated with plasma in water, saline, and YPD, compared to that in Ar gas treated cells. Yeast cells were treated with plasma and Ar gas for 3 min. Relative absorbance was calculated as relative absorbance (%) = (absorbance at 490 nm in plasma treated cells/absorbance at 490 nm in Ar gas treated cells)×100. Each value is the average of three technical replicates.

The viability of the yeast cells was assessed by the ratio in CFU number (indicated as percentage) between the plasma and Ar gas (control) treatments. The relative viability of yeast cells treated with plasma (compared to control treatment) decreased considerably over exposure time in water and saline, but did not change significantly in YPD media (Fig. 1A). When the cells were treated in water, most of the cells were dead after the 120 second exposure. However, yeast cells treated in saline were inactivated less severely than in water. Cells in YPD media were not much affected by the 3 min plasma treatment (Fig. 1A).

Cell viability was also assessed by the MTT assay. Mitochondrial dehydrogenase in viable cells reduces MTT (yellow color) to MTT formazan (purple color), and this enzyme is inactive in dead cells. Thus, cell viability can be assessed by measuring the level of MTT formazan product (purple). Compared to the control (Ar gas treatment), formazan production was reduced more than 50% in the plasma-treated cells in water indicating that more than 50% of cells were inactive (Fig. 1B). An approximate 20% decrease in cell activity was observed in saline following the plasma treatment, and there was no significant change in active cell numbers between plasma and Ar gas treatment in YPD (Fig. 1B).

**Figure 2:**
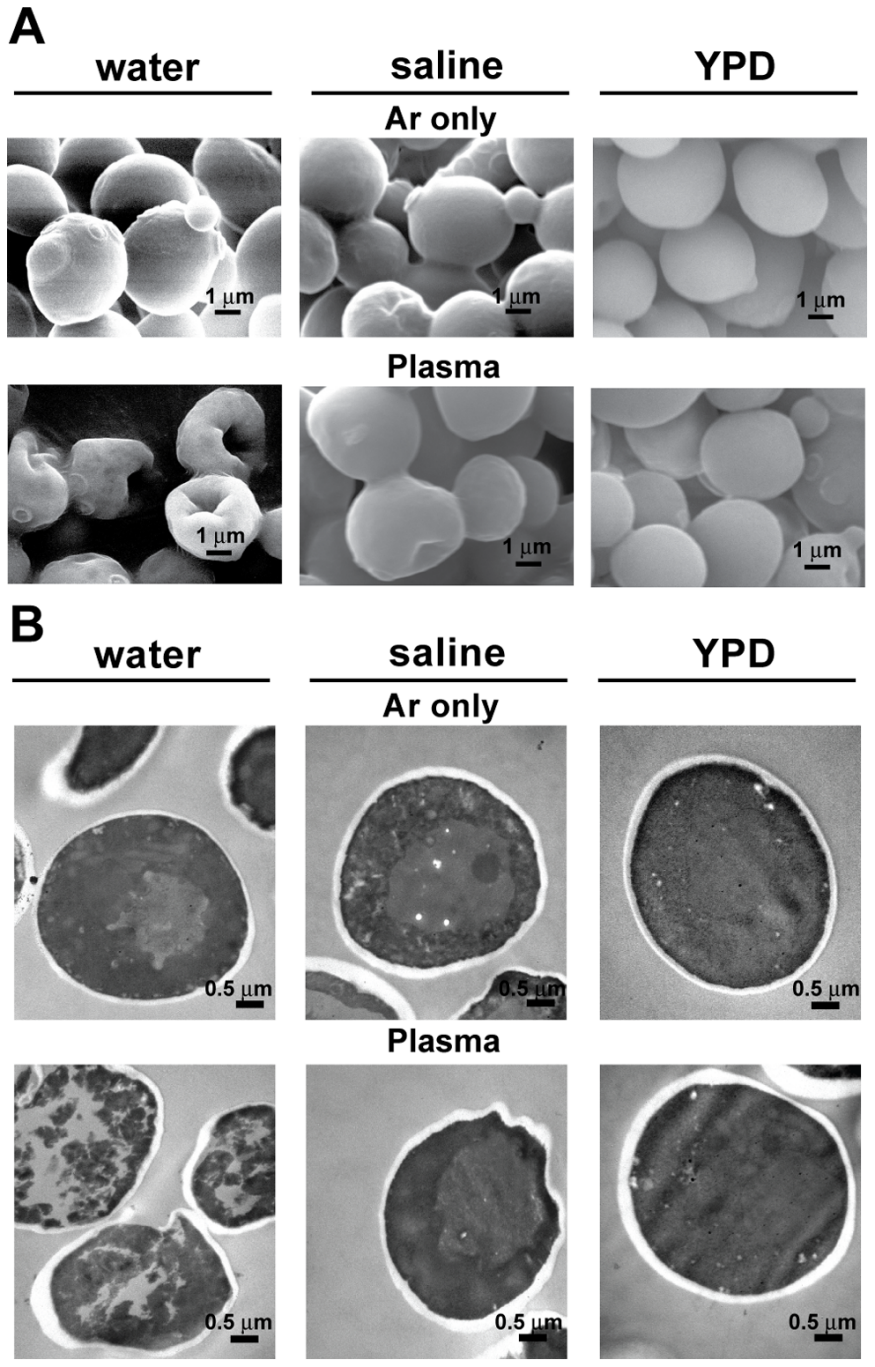
Morphology and internal structure of yeast cells after plasma treatment. (**A**) Cells analyzed by scanning electron microscopy. (**B**) Cells analyzed by transmission electron microscopy. Yeast cells in water, saline, and YPD were exposed to Ar gas and plasma for 3 min and used for the microscopic analysis.

The SEM analysis showed that the majority of yeast cells in water was severely crushed and shrunken after plasma treatment for 3 min (Fig. 2A). Yeast cells exposed to only Ar gas for 3 min in water did not exhibit any significant changes in morphology and were round in shape with a smooth surface, similar to the wild type (Fig. 2A). When the cells were treated in saline, a high proportion of cells was unaffected, but some cells were shrunken and crushed (Fig. 2A). Compared to saline, most cells treated in YPD media did not exhibit any obvious changes in morphology (Fig. 2A).

In the TEM analysis on the internal structure of yeast cells, the internal area of cells treated with plasma in water was much less dense compared to that in cells treated in saline or YPD media (Fig. 2B). Empty spaces were more often observed in the yeast cells treated in water than in the cells treated in saline or YPD (Fig. 2B). When yeasts were treated in saline, the majority of cells appeared similar to the control (treated with Ar gas), but cells with a electro-sparse intracellular structure were also observed (data not shown).

### Effects of Plasma on Lipids and Genomic DNA in Yeast Cells

**Figure 3:**
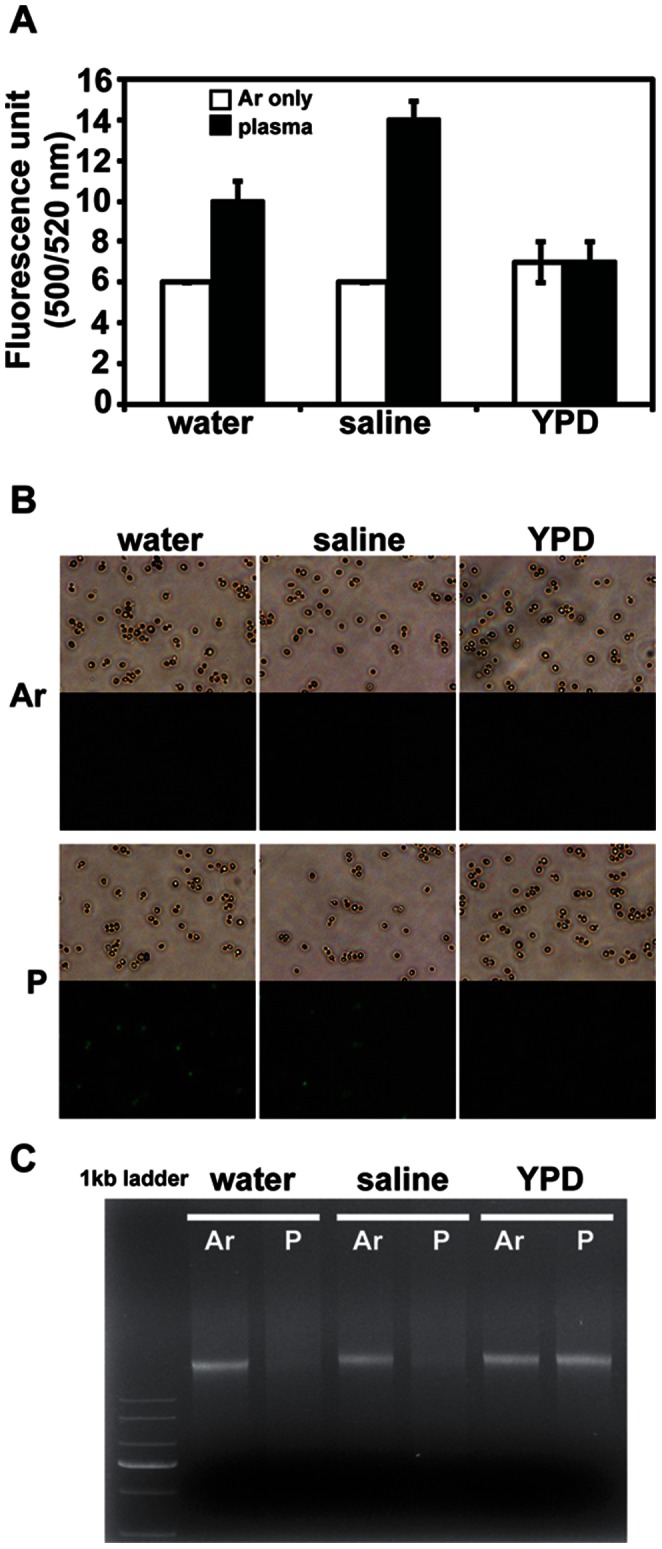
Lipid peroxidation and genomic DNA stability analyses. Yeast cells (2×10^7^) were exposed to Ar gas and plasma for 3 min and used for the analyses of lipid peroxidation and genomic DNA. (**A**) Production of 4-hydroxynonenal (4-HNE) measured as fluorescence intensity at 500/520 nm in cells treated with plasma in water, saline, and YPD. Each value is the average of three technical replicates. (**B**) Fluorescence image of cells after Ar gas and plasma treatment in water, saline, and YPD. (**C**) Agarose gel electrophoresis of genomic DNA. Same amount of genomic DNA (2 µg) was loaded on the 1% agarose gel.

Lipid peroxidation and stability of genomic DNA were examined to analyze the effects of plasma on the cell at the molecular level. Production of 4-HNE was monitored as an indication of membrane lipid peroxidation. The increased level of 4-HNE detected by fluorescence (500/522 nm)-labeled antibody was observed in the cells treated with plasma in water and saline, but not those in YPD (Fig. 3A). Many cells exhibited fluorescence in water and saline when observed under a fluorescence microscope (Fig. 3B). Although basal fluorescence was detected in cells exposed to only Ar gas, fluorescence intensity increased after plasma exposure only in cells submerged in water and saline (Fig. 3A and B) indicating that cells in water and saline underwent more severe lipid peroxidation.

When the same amount of genomic DNA (2 µg) extracted from the treated cells was examined on an agarose gel, the intensity of the genomic DNA bands decreased significantly after plasma exposure in water and saline (Fig. 3C). However, no significant difference in DNA band intensity was observed between the plasma and Ar gas (control) treatments in YPD media (Fig. 3C).

### *HOG1* MAP Kinase is Phosphorylated in Response to Plasma Treatment

**Figure 4:**
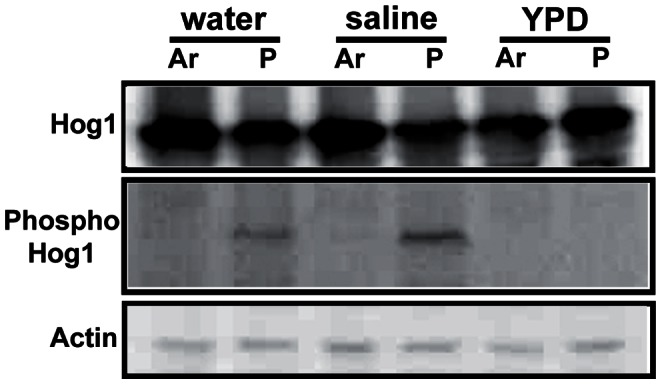
Phosphorylation of *HOG1* MAP kinase after Ar gas and plasma treatment analyzed by Western blot. The levels of Hog1p expression and phosphorylation are shown in the first and second panel, respectively. The third panel shows the Western blot for actin as loading control. Total protein was extracted from the cells treated with Ar gas and plasma in water, saline, and YPD for 3 min, and run on a 12% SDS-PAGE (sodium dodecyl sulfate polyacrylamide gel electrophoresis) gel.

Because the majority of components in Ar plasma is ROS and RNS, yeast cells will encounter severe oxidative stress and this will induce various defense responses. One of the responses could be activation of MAP kinase. The MAP kinase signaling pathway is involved in stress regulation in yeasts and fungi [28]. We first examined the activation (phosphorylation) of three major MAP kinases in *S. cerevisiae* Fus3p, Hog1p, and Slt2p after plasma treatment to understand the molecular responses of yeast to plasma stress in different background solutions. Phosphorylation of Fus3p and Slt2p MAP kinases was not observed in cells after plasma treatment in any of the background solutions (data not shown), whereas *HOG1* MAP kinase was phosphorylated in the cells treated with plasma only in water and saline (Fig. 4). No remarkable difference in phosphorylation level was observed between water and saline treatment. The level of *HOG1* protein expressed was not significantly different between control (Ar gas only treated) and plasma treated cells in all background solutions, indicating that the plasma did not affect protein expression level but did affect the level of protein phosphorylation (Fig. 4). Plasma exposure in YPD did not trigger activation of *HOG1* MAP kinase (Fig. 4).

### The Efficiency of Yeast Inactivation by Plasma is Affected by pH and Salt Strength of the Background Solution

**Figure 5:**
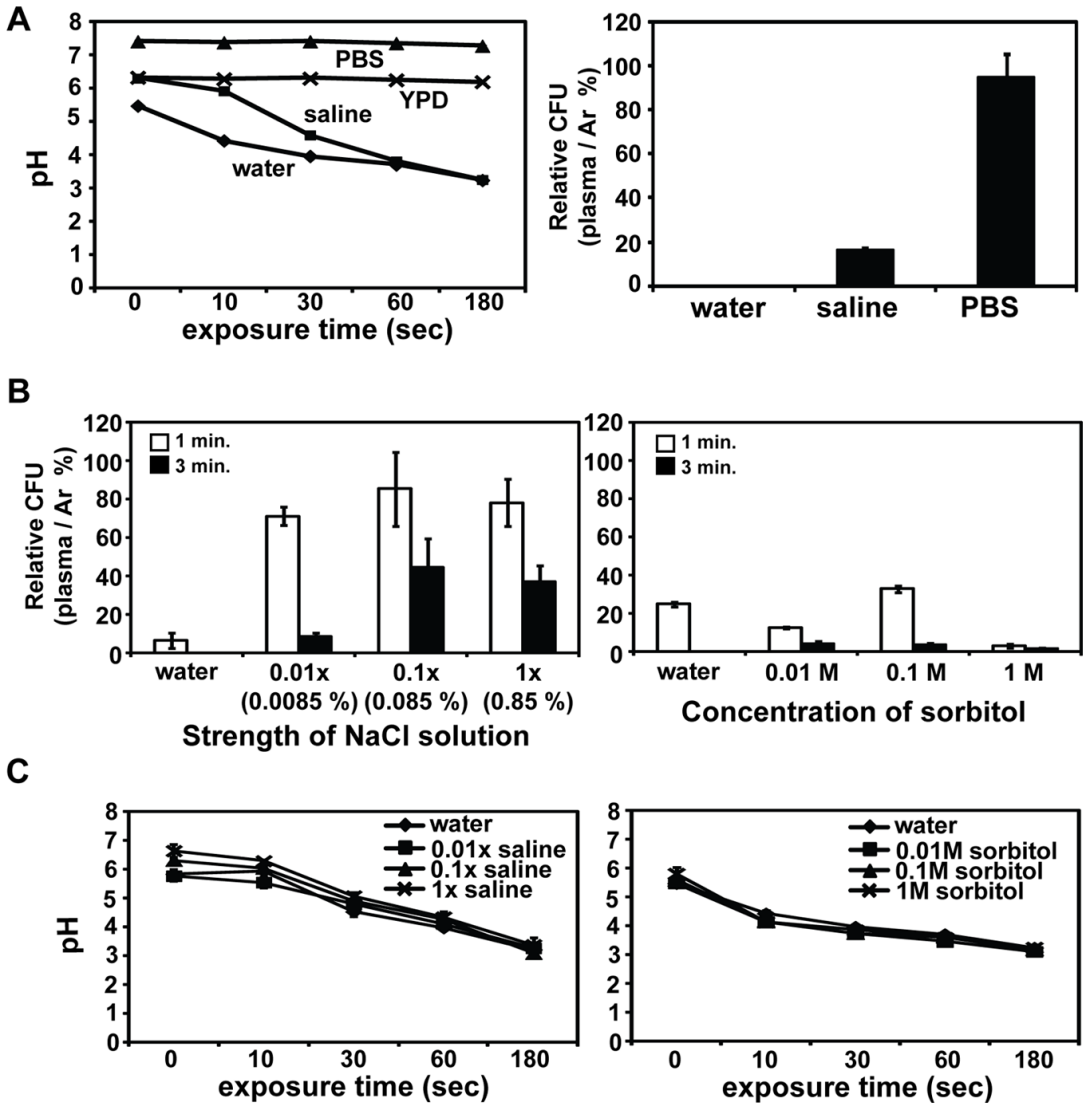
pH change after plasma treatment and effects of osmotic strength of background solutions. (**A**) pH of water, saline, PBS, and YPD after plasma treatment (left panel) and the effect of PBS on yeast cell viability (right panel). For pH measurements, 1 ml of each solution was exposed to plasma for the indicated time. The effect of PBS was assessed by measuring relative viability of plasma-treated cells compared to that of Ar gas-treated cells. Cells were treated with plasma for 3 min. (**B**) Viability of yeast cells in different concentrations of NaCl (left panel) and sorbitol (right panel) after plasma treatment. Yeast cells were treated with Ar gas and plasma for 1 and 3 min. (**C**) Changes in pH of NaCl (left panel) and sorbitol (right panel) solutions during plasma treatment. One ml of saline and sorbitol was exposed to plasma at the indicated concentrations for 10, 30, 60, and 180 sec. In (A) and (B), cell viability was assessed by relative ratio in colony forming units (CFU) calculated as follows: relative CFU (%) = (CFU number of plasma treated yeast/CFU number of Ar gas treated yeast)×100. All values are the average of three technical replicates.

We investigated the influence of pH and osmotic strength of the background solution on cells to determine factors causing different cell responses in water, saline, and YPD upon plasma exposure. Plasma treatment rapidly decreases the pH of water and the acidity of water produces toxic effects on cells [15], [20]. pH of both water and saline decreased during plasma exposure in a time-dependent manner and reached about 3 after a 3 min exposure (Fig. 5A, left panel). In contrast, the pH of YPD did not change as much from the initial value of 6–7 during plasma treatment (Fig. 5A, left panel). Cell damage observed in water and saline was likely caused by acidification of the solutions by plasma. To further examine this notion, we treated yeast cells in PBS and found that the majority of cells survived after plasma treatment and that the pH of PBS was maintained between 7 and 8 during plasma exposure (Fig. 5A, left and right panel).

Yeast cells in saline showed a higher survival rate than those in water as shown in Fig. 1A. Additional factor(s) in saline may have modulated the plasma effect on yeast cells. To test this hypothesis, we first examined the influence of osmotic strength of the saline on plasma action by using differently diluted saline solutions. Yeast cells were more severely impaired in viability when treated by plasma in 100 times diluted saline solution (0.01×, hypotonic) compared to undiluted and 10 times diluted solutions (Fig. 5B, left panel). However, when cells were treated in different concentrations of sorbitol, a well known osmotic stabilizer, a low survival rate was observed at all concentrations (Fig. 5B, right panel). This seemed to be due to the decrease in pH of sorbitol by plasma (Fig. 5C, right panel). Both saline and sorbitol at all concentrations became acidic following the plasma treatment (Fig. 5C) but only undiluted saline resulted in increased survival of yeast cells. This indicates that salt strength may have played a role in protecting the yeast cells from plasma stress.

### The Level of Reactive Species is Different on the Solution Surface, and Inside the Solution and Cells

**Figure 6:**
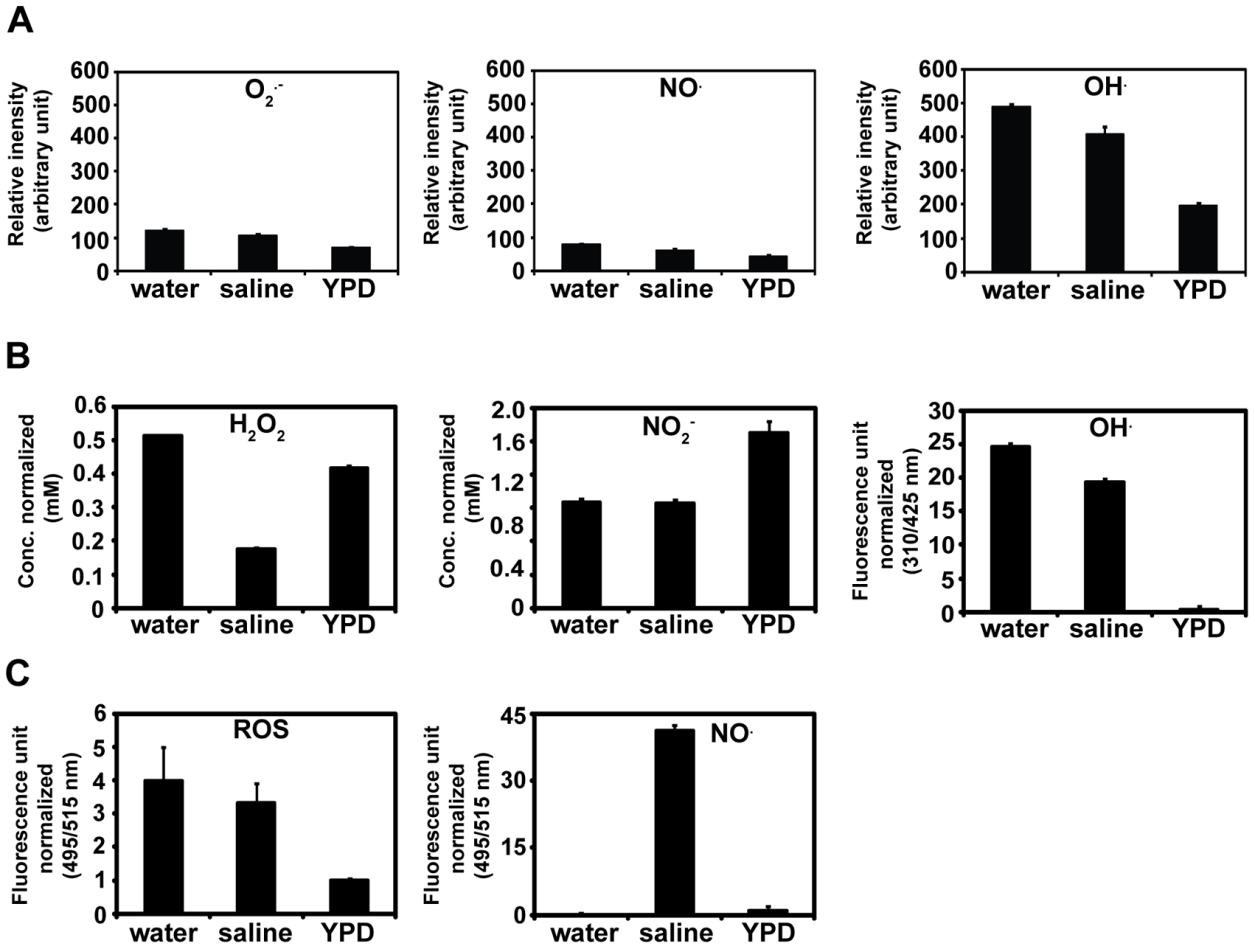
The level of reactive oxygen species (ROS) and reactive nitrogen species (RNS). ROS and RNS levels were measured on the surface of solutions (**A**), and inside solutions (**B**) and cells (**C**) after Ar gas and plasma treatments. Absorbance values in (A) were measured by optimal emission spectroscopy and are the average of five replicates. In (B) and (C), all values are normalized, in which the level measured after Ar gas treatment was subtracted from that measured after plasma treatment. Each value is the average of three technical replicates.

It may be possible that the level and composition of ROS and RNS from plasma is affected through the interaction between plasma and the surrounding environment. We showed previously that the Ar plasma jet used in this study produces OH radical, NO, and superoxide as major reactive species [21]. The optical emission spectrum profile analyzed on the surface of three different solutions (water, saline, YPD) showed that the OH radical was the most highly detected, compared to other reactive species such as superoxide and NO (Fig. 6A). The relative levels of OH radical, superoxide, and NO were higher on the surface of water than that of saline and YPD, and the lowest level of three species was observed on the YPD surface (Fig. 6A).

The OH radical was detected relatively higher inside the water than inside the saline or YPD, and the level of OH radical in saline was higher than YPD (Fig. 6B). The OH radical was hardly detected in YPD (Fig. 6B). The H_2_O_2_ concentration measured after plasma treatment was higher in water than that in saline or YPD, and a much lower concentration was observed in saline (Fig. 6B). This may be because H_2_O_2_ is used in the reaction with NaCl to generate NaOCl. The NO level, estimated as the concentration of NO_2_^−^, was similar in water and saline after plasma treatment (Fig. 6B). The concentration of NO_2_^−^ was higher in YPD treated with plasma (Fig. 6B), probably because NO_3_^−^ derived from nitrogen nutrient sources in the YPD was detected together by the NO assay kit.

**Figure 7:**
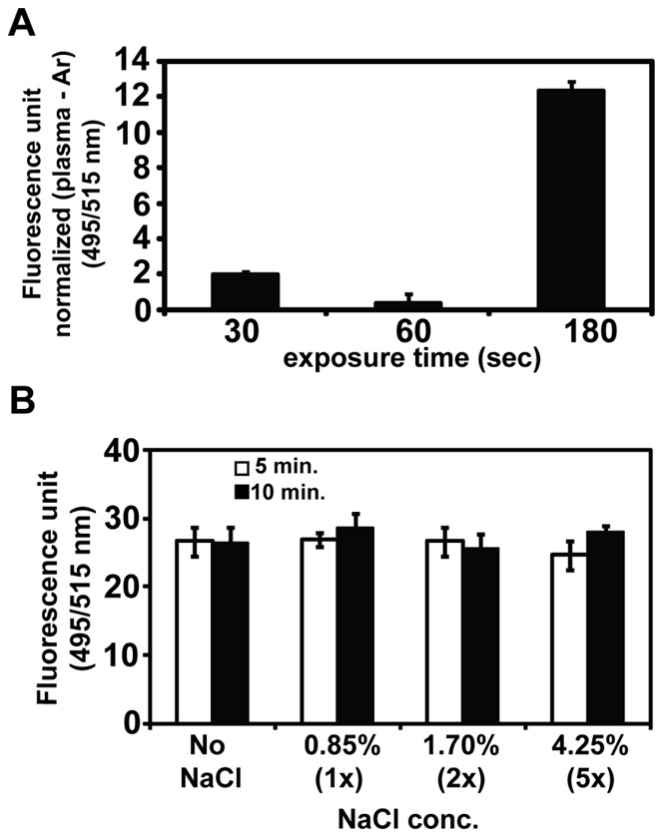
Intracellular level of NO during plasma exposure time and in different concentrations of NaCl. (**A**) Intracellular NO level was measured after plasma exposure in saline for 30, 60, and 180 sec. Fluorescence values are normalized, in which the level measured after Ar gas treatment was subtracted from that measured after plasma treatment. (**B**) NO level was measured in cells incubated in different concentrations of NaCl solution for 5 and 10 min. Each value is the average of three technical replicates.

Intracellular ROS and NO levels were estimated using fluorescent probes. The ROS level detected by H_2_DCFDA was dramatically higher in the plasma-treated cells in water and saline than that in YPD, and it was higher in water than in saline (Fig. 6C). Intracellular ROS level among the three solutions showed a similar pattern as the level of OH radical inside the solutions. Interestingly, the intracellular NO level increased significantly in cells treated with plasma only in saline (Fig. 6C). In water and YPD, a background level of NO was measured in the plasma-treated cells (Fig. 6C). The increased intracellular NO level in saline was dependent on plasma exposure time (Fig. 7A), indicating that NO may be endogenously generated. In plants, the endogenous synthesis of NO is occasionally induced by salt stress [29]. To examine the effect of salt stress on NO generation in yeast cells, NO was measured in cells incubated in a higher concentration of NaCl. No significant difference in intracellular NO level was observed between control (no NaCl) and all concentrations of NaCl solutions, indicating that the NO detected in yeast cells might be related to the plasma treatment (Fig. 7B).

## Discussion

### Plasma Produces Various Outcomes During Microbial Inactivation

Our study clearly demonstrates that non-thermal atmospheric pressure plasma generated different level of lethal effects on microorganisms through interaction with surrounding environments. Several studies have shown that sterilization efficacy of non-thermal plasma can be regulated by changing physical conditions of plasma discharge such as oxygen gas level, gas flow, and electrical power [9]–[11], [30]. However, environmental influences have not been frequently reported. A recent study demonstrated that the bactericidal effects of plasma in water and saline increase due to the acidification of solutions by air plasma and that PBS exposed to N_2_ plasma becomes highly toxic to bacteria [18].

Our results suggest that controlling environmental factors, particularly surrounding media, can be a way to increase sterilization efficiency by plasma. In our study, water created the most detrimental environment to yeast cells during plasma exposure. Other study also showed that increased humidity improves killing efficiency of microorganisms [31]. In our study, yeast cells in saline were also severely damaged at the cellular and molecular level. This indicates that plasma can also be effective for inactivating microbes in biological fluids because most biological fluids contain NaCl. However, plasma treatment of YPD media containing various nutrients produced the least harmful effect on yeast cells. Similar results were observed in a previous study using the fungus *Neurospora crassa;* fungal spores were much less damaged in fungal nutrient media under plasma exposure [21]. These results suggest that microbes that infect cells (a soup of various nutrient molecules) are more difficult to be eradicated by plasma.

### Interaction between Plasma and the Environment May Affect Sterilization Efficiency

As demonstrated in our results, yeast cells were dramatically damaged in water and saline by plasma treatment but not in YPD. A main reason for this might be acidification of water and saline by plasma because most yeast cells were alive in PBS, and YPD maintained a constant neutral pH during plasma treatment. Acidification of water by plasma is mostly due to the production of nitric acid originating from NO produced by plasma [21], and this may be the case in our results. However, our results also reveal that pH was not the only factor affecting cell performance under plasma attack in saline. Several studies have demonstrated that pH changes in media are not the predominant reason for microbial death [21], [32]. In our results, more yeast cells in saline survived and showed fewer morphological changes compared to those in water under plasma attack. It seemed that salt strength in the saline was closely related to the higher survival of yeast cells rather than osmotic balance between cells and saline. Several results in our study support this notion. First, diluting the saline resulted in a significant reduction in yeast cell viability, although there was no change in pH (acidic) between diluted and undiluted saline (Fig. 5B and C). Second, adding sorbitol, an osmotic stabilizer, to water did not improve survival of yeast cells exposed to plasma (Fig. 5B). Our findings demonstrate that buffering capacity and salt strength of surrounding media are very critical for determining the efficiency of plasma to inactivate microbes.

The effect of surrounding media on composition of reactive species from plasma is also important when inactivating yeast cells. From our results, it seemed that the OH radical was the major species that has caused the differential death rates of yeast cells in water, saline, and YPD. This is because the overall level of OH radicals was higher on the solution surface than the other two species, superoxide and nitric oxide, and its level was highest on the water surface. Additionally, the level of OH radicals was the highest inside water and the lowest in YPD. OH radicals may be quenched when plasma passes through saline and YPD solutions, eventually leading to less cell damage in saline and YPD. The toxic effects of OH radicals in plasma have already been observed in other studies [33]–[34]. OH radicals seems to be involved in membrane lipid peroxidation. Our data showed that the level of lipid peroxidation increased in cells treated in water and saline. Cell membranes may be damaged by lipid peroxidation probably due to the action of OH radicals, and increased membrane permeability will break ion balance between inside and outside the cell. This will lead to an increase in osmotic pressure to the cell and eventually induce activation of osmoregulatory mechanisms in the cell. As shown in our results, *HOG1* MAP kinase regulating osmotic stress was activated in water and saline during plasma exposure.

It is not clear if the OH radical was directly involved in damaging yeast cells or it indirectly affected the cells by its involvement in the production of H_2_O_2_ or other toxic molecules inside the solutions. Our data show that the level of H_2_O_2_ and NO inside the three solutions did not match well with differences in cell viability. Thus, H_2_O_2_ and NO levels inside the solutions may not have been critical for differential inactivation of yeast cells. Another role of the OH radical inside solutions is that it contributed to building up intracellular ROS, eventually leading to cell damage.

Interestingly, our results suggest that plasma treatment in saline has induced endogenous NO production in yeast cells. Salt stress on yeast cells does not trigger intracellular NO production, as demonstrated in our study. The concentration of NaCl in saline (0.85%) was not sufficient to generate salt stress in yeast cells and our results show no significant changes in intracellular NO level during incubation at higher concentration of NaCl (up to 4.25%).

In summary, the efficiency of plasma to inactivate yeast was affected by the interaction between plasma and the surrounding environments, the resistance of the surrounding media to pH change by plasma, modulation of the plasma effect by salt concentration in surrounding media, and alterations in reactive species through the interaction of plasma with the surrounding media.
